# Clinical predictors and differential diagnosis of posterior reversible encephalopathy syndrome

**DOI:** 10.1007/s13760-017-0750-6

**Published:** 2017-01-31

**Authors:** Laetitia della Faille, S. Fieuws, W. Van Paesschen

**Affiliations:** 1 Division of Neurology, University Hospitals Leuven, KU Leuven, University of Leuven, Louvain, Belgium; 2 Interuniversity Institute for Biostatistics and Statistical Bioinformatics, KU Leuven and Hasselt University, Leuven, Belgium

**Keywords:** PRES, Posterior reversible encephalopathy syndrome, Imaging, Clinical predictors, Diagnosis

## Abstract

The aim of our study is to determine the clinical predictors and the differential diagnosis of posterior reversible encephalopathy syndrome (PRES) in patients presenting with acute neurological symptoms and risk factors for PRES. Using the diagnostic algorithm for PRES from Fugate and Rabinstein (Lancet Neurol 14(9):914–925, 1), we carried out a retrospective study on 220 patients, presenting with acute neurological symptoms such as seizures, encephalopathy, headache, visual disturbances or other focal neurological signs that appear in the clinical setting of risk factors such as hypertension/blood pressure fluctuations, chemotherapy, renal failure, autoimmune disorders, or eclampsia, in whom imaging of the brain was performed to exclude PRES. Seventeen percent of patients had a radiologically confirmed diagnosis of PRES. Univariable logistic regression showed a significant association between PRES and epileptic seizures, encephalopathy, hypertension, chemotherapy and renal failure. Multivariable logistic regression of acute neurological symptoms and risk factors showed a significant association of epileptic seizures, encephalopathy, visual disturbances, hypertension and chemotherapy with PRES. Using these variables to predict PRES yielded a discriminative ability (AUC) equal to 0.793. Diagnoses when PRES was not confirmed included primary or secondary headaches (26%), toxic-metabolic encephalopathy (21%), vascular pathology (12%) and other less frequent disorders. Epileptic seizures, encephalopathy, visual disturbances, hypertension, renal failure and chemotherapy were the best clinical predictors of PRES, while headache, immune suppression and autoimmune disease were not useful for the clinical diagnosis of PRES in our study.

**Electronic supplementary material:**

The online version of this article (doi:10.1007/s13760-017-0750-6) contains supplementary material, which is available to authorized users.

## Introduction

Posterior reversible encephalopathy syndrome (PRES) has been increasingly recognized during the past two decades. It was first described by Hinchey et al. as a reversible, predominantly posterior leukoencephalopathy [2]. This disorder is characterized by acute neurological symptoms, such as seizures, encephalopathy, headache, visual disturbances or other focal neurological signs that appear in the clinical setting of risk factors, such as hypertension or blood pressure fluctuations, cytotoxic drugs, renal failure, autoimmune disorders and eclampsia [1]. The diagnosis is confirmed by demonstrating subcortical vasogenic brain oedema on brain imaging. In this retrospective study, our aim was to study the predictive value of these acute neurological symptoms and risk factors in the diagnosis of PRES, and to establish the spectrum of differential diagnoses when PRES was not confirmed on cerebral imaging.

## Methods

We retrospectively analysed digital medical files of patients seen in the Department of Neurology in the University Hospital Gasthuisberg, Leuven, Belgium, from January 2012 to December 2015. We performed a search using the key words “Posterior reversible encephalopathy syndrome” or “PRES” in the differential diagnosis of an acute neurological illness. In order to ensure an objective and unambiguous selection of patients with PRES among these patients, we considered only patients with reversible vasogenic oedema on brain imaging (i.e. radiologically confirmed diagnosis of PRES) as having PRES, while patients without confirmation on brain imaging were considered as not having PRES. Based on the radiological patterns described by Bartynski and Boardman [3], we classified the radiological findings on brain imaging into typical and atypical PRES patterns.

We performed a detailed review of their clinical information. We assessed more specifically demographics, typical symptoms (seizures, encephalopathy, headache, visual disturbances or other focal neurological signs) and risk factors for PRES (hypertension, cytotoxic drugs, renal failure, autoimmune disorders, and pre-eclampsia or eclampsia), as described by Fugate and Rabinstein [1]. Focal neurological signs were defined as deficits from the central or peripheral nervous system. In our study, hypertension was defined as a systolic blood pressure above 140 mmHg or a diastolic blood pressure higher than 90 mmHg, and renal failure as an estimated glomerular filtration rate (eGFR) less than 60 mL/min. Outcome of patients with PRES was measured at discharge from the hospital, using the Glasgow Outcome Scale (GOS), ranging from 1 (death) to 5 (good recovery). Since several patients had important premorbid disabilities, we compared the outcome of all patients with their premorbid status. Finally, we noted the differential diagnosis in patients without PRES.

Fisher’s exact test was used to compare the percentage of patients with PRES as a function of the presence or absence of these acute neurological symptoms and risk factors. The distribution of the number of symptoms and the number of risk factors was compared between patients with and without PRES using a Mann–Whitney *U* test. Odds ratio from univariable logistic regressions is reported. The discriminative ability of each variable was evaluated with the area under the operating characteristic curve (AUC), where value 0.5 equals random prediction and value 1 perfect discrimination. A multivariable logistic regression model was applied using a backward selection procedure with *p* = 0.157 as critical *p* value to stay in the model. The use of this critical value corresponds to using the Akaike Information Criterion (AIC) for model selection. With AIC, we required that the increase in model *χ* ^2^ had to be larger than two times the degrees of freedom [4]. The AUC of the final multivariable logistic regression model is reported. Since the same data were used to build and to evaluate the model, the AUC was corrected for overoptimism using an advanced bootstrap procedure [5]. All analyses have been performed using SAS software, version 9.2 of the SAS System for Windows.

## Results

Two hundred and twenty medical files of patients presenting with an acute neurological illness, in whom PRES was considered as a possible diagnosis, were selected. There were 134 women and 86 men, age ranging from 14 to 90 years. Thirty-seven (17%) had the diagnosis of PRES confirmed on brain imaging (CT or MRI). The age of the radiologically confirmed PRES patients ranged from 16 to 85 years, with a median age of 49 years. Among these, there were 26 females (70%) and 11 males (30%).

Among the 37 patients with PRES, 22 (59%) had epileptic seizures, 21 (57%) encephalopathy, ranging from drowsiness to coma, 16 (43%) had focal neurological deficits, 13 (35%) had visual disturbances (decreased visual acuity, visual field deficits, cortical blindness, or visual hallucinations) and 13 (35%) had headache. Results are shown in Table 1.

**Table 1:** Acute neurological symptoms in patients with and without PRES

Variable	Not PRES (*n* = 183)	Radiologically confirmed PRES (*n* = 37)	*p* value
Epileptic seizures			<0.001
No	153/183 (84%)	15/37 (41%)	
Yes	30/183 (16%)	22/37 (59%)	
Encephalopathy			0.001
No	132/183 (72%)	16/37 (43%)	
Yes	51/183 (28%)	21/37 (57%)	
Focal neurological deficits			0.254
No	124/183 (68%)	21/37 (57%)	
Yes	59/183 (32%)	16/37 (43%)	
Headache			0.367
No	103/183 (56%)	24/37 (65%)	
Yes	80/183 (44%)	13/37 (35%)	
Visual disturbances			0.433
No	131/183 (72%)	24/37 (65%)	
Yes	52/183 (28%)	13/37 (35%)	
Number of symptoms			<0.001
0	12/183 (7%)	0/37 (0%)	
1	89/183 (49%)	6/37 (16%)	
2	65/183 (36%)	17/37 (46%)	
3	15/183 (8%)	11/37 (30%)	
4	2/183 (1%)	3/37 (8%)	
Number of symptoms			<0.001
Median	1.0	2.0	
Range	(0.0; 4.0)	(1.0; 4.0)	

Variables presented with percentages are analysed using a Fisher’s exact test. Variables summarized by medians are analysed using a Mann–Whitney *U* test. All reported *p* values are two-sided

Thirty-one (84%) patients with PRES in our study had hypertension as a risk factor, 20 (54%) had renal failure, 11 (30%) immune suppression, 12 (32%) received chemotherapy, 6 (16%) had an autoimmune disease and 1 (3%) had eclampsia. Results are shown in Table 2.

**Table 2:** Risk factors in patients with and without PRES

Variable		Not PRES (*n* = 183)	Radiologically confirmed PRES (*n* = 37)	*p* value
Hypertension				<0.001
No		87/183 (48%)	6/37 (16%)	
Yes		96/183 (52%)	31/37 (84%)	
Chemotherapy				0.019
No		155/183 (85%)	25/37 (68%)	
Yes		28/183 (15%)	12/37 (32%)	
Renal failure				0.044
No		117/183 (64%)	17/37 (46%)	
Yes		66/183 (36%)	20/37 (54%)	
Immune suppression				0.146
No		104/183 (57%)	26/37 (70%)	
Yes		79/183 (43%)	11/37 (30%)	
Eclampsia				0.168
No		183/183 (100%)	36/37 (97%)	
Yes		0/183 (0%)	1/37 (3%)	
Pre-eclampsia				0.219
No		173/183 (95%)	37/37 (100%)	
Yes		10/183 (5%)	0/37 (0%)	
Autoimmune disorders				0.615
No		158/183 (86%)	31/37 (84%)	
Yes		25/183 (14%)	6/37 (16%)	
Number of risk factors				0.029
0		15/183 (8%)	0/37 (0%)	
1		70/183 (38%)	10/37 (27%)	
2		64/183 (35%)	13/37 (35%)	
3		30/183 (16%)	11/37 (30%)	
4		4/183 (2%)	3/37 (8%)	
Number of risk factors				0.003
Median		2.0	2.0	
Range		(0.0; 4.0)	(1.0; 4.0)	

Variables presented with percentages are analysed using a Fisher’s exact test. Variables summarized by medians are analysed using a Mann–Whitney *U* test. All reported *p* values are two-sided

Univariable logistic regressions of acute neurological symptoms showed a significant association between PRES and epileptic seizures [odds ratio (OR) 7.48, *p* < 0.0001], encephalopathy (OR 3.40; *p* = 0.0010) and number of symptoms (OR 3.17; *p* < 0.0001). Results are shown in Table 3.

**Table 3:** Acute neurological symptoms and risk factors as predictors of PRES: univariable and multivariable logistic regressions

Variable symptoms	Univariable associations			Multivariable associations	
	OR (95% CI)	*p* value	AUC (95% CI)	OR (95% CI)	*p* value
Epileptic seizures	7.48 (3.48; 16.06)	<0.0001	0.72 (0.63; 0.80)	6.93 (2.86; 16.79)	<0.0001
Encephalopathy	3.40 (1.64; 7.02)	0.0010	0.64 (0.56; 0.73)	3.35 (1.36; 8.25)	0.0085
Focal neurological deficits	1.60 (0.78; 3.29)	0.2001	0.56 (0.47; 0.64)	–	–
Headache	0.70 (0.33; 1.46)	0.3369	0.54 (0.46; 0.63)	–	–
Visual disturbances	1.37 (0.65; 2.88)	0.4149	0.53 (0.45; 0.62)	2.62 (1.02; 6.75)	0.0457
Increasing number of symptoms	3.17 (1.99; 5.05)	<0.0001	0.75 (0.67; 0.83)		
Risk factors					
Hypertension	4.68 (1.86; 11.76)	0.0010	0.66 (0.59; 0.73)	5.89 (2.07; 16.77)	0.0009
Chemotherapy	2.66 (1.20; 5.90)	0.0163	0.59 (0.51; 0.67)	3.04 (1.14; 8.12)	0.0265
Renal failure	2.09 (1.02; 4.26)	0.0435	0.59 (0.50; 0.68)	–	–
Immune suppression	0.56 (0.26; 1.20)	0.1329	0.57 (0.48; 0.65)	–	–
Pre-eclampsia	ND	0.0519	0.53 (0.51; 0.54)	–	–
Autoimmune disorders	1.22 (0.46; 3.23)	0.6841	0.51 (0.45; 0.58)	–	–
Increasing number of risk factors	1.82 (1.23; 2.68)	0.0025	0.65 (0.55; 0.74)		

The optimism-corrected AUC of the multivariable prediction model based on the advanced bootstrap equals 0.792. ND: odds ratio is not defined due to the absence of patients with PRES in the pre-eclampsia group

*OR* odds ratio, *CI* confidence interval, *AUC* area under the operating characteristic curve

Univariable logistic regressions of the risk factors showed a significant association between hypertension (OR 4.68; *p* = 0.0010), chemotherapy (OR 2.66; *p* = 0.0163), renal failure (OR 2.09; *p* = 0.0435) and an increasing number of risk factors (OR 1.82; *p* = 0.0025) with PRES. Only three patients (8%) with PRES had none of these three risk factors. The factor eclampsia was not considered, since there was only a single patient with this risk factor. Results are shown in Table 3.

Multivariable logistic regression of acute neurological symptoms and risk factors showed a significant association between epileptic seizures (OR 6.933; *p* < 0.0001), encephalopathy (OR 3.35; *p* = 0.0085), visual disturbances (OR 2.62; *p* = 0.0457), hypertension (OR 5.89; *p* = 0.0009) and chemotherapy (OR 3.04; *p* = 0.0265) with PRES. Results are shown in Table 3. The discriminative ability (AUC) of this multivariable model equaled 0.793, hence using this set of predictors a moderate to good prediction of PRES can be expected.

From the 220 patients in our study, 183 did not have radiologically confirmed PRES. The 183 patients without radiologically confirmed PRES had headache (26%), toxic-metabolic encephalopathy (21%), vascular pathology (12%), infectious pathology (8%), epileptic seizures (8%), neurodegenerative diseases (4%), psychiatric disease (4%), tumours (4%), ophthalmological illness (3%), inflammatory disorders (2%), intracranial hypertension (2%), other diagnoses (3%) and unknown diagnoses (2%). Notice that the clinical diagnosis of PRES was made without brain imaging confirmation in three patients (2%) (Table 5—supplemental).

**Fig. 1:**
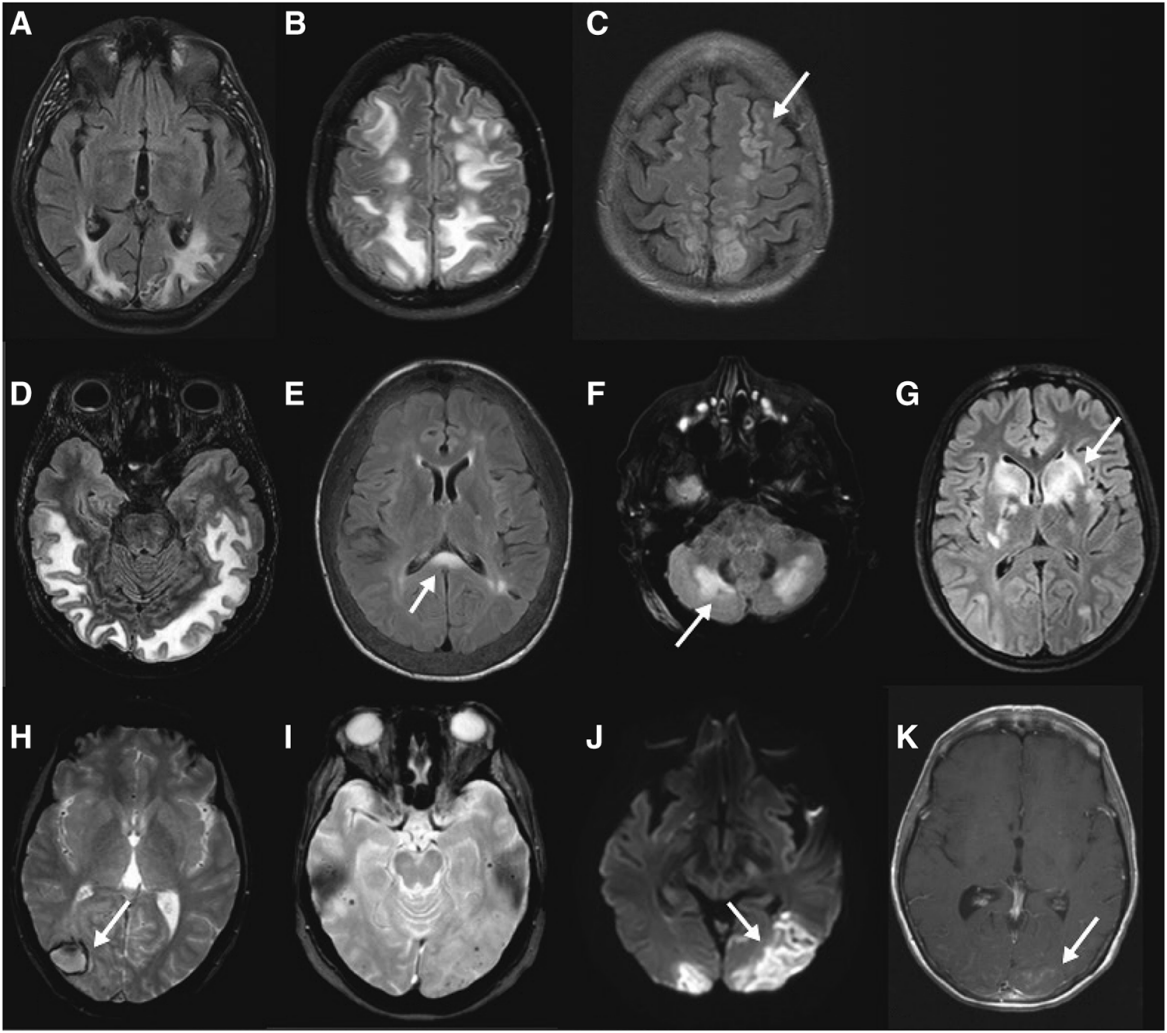
PRES patterns on MRI brain imaging. **a** FLAIR dominant parieto-occipital pattern, **b** FLAIR holohemispheric watershed pattern, **c** FLAIR sulcus frontalis superior (*arrow*) and holohemispheric watershed pattern, **d** temporal lobe pattern, **e** splenium corporis callosi pattern, **f** cerebellar pattern, **g** basal ganglia pattern. **h** GE (gradient echo) macrohaemorrhage, **i** GE multiple microhaemorrhages, **j** diffusion-weighted restricted diffusion, **k** contrast enhancement

Thirty-five patients had lesions on MRI that were compatible with PRES (28 typical PRES patterns and 7 atypical PRES patterns) [3]. One patient could not undergo MR imaging, but CT imaging was compatible with PRES. Another patient had typical parieto-occipital oedema on CT imaging, which had disappeared on MRI performed a few weeks later. Brain MRI most frequently showed dominant lesions in a typical parieto-occipital pattern (66%). The holohemispheric watershed pattern was the second most frequent lesions, present in 14% of patients. The third most frequent pattern in our study was the cerebellar pattern in 9% of patients. No patient had a dominant superior frontal sulcus pattern. Less typical patterns were seen as well, such as basal ganglia or brainstem lesions, or lesions in the splenium corporis callosi. Results are shown in Table 4. Images are shown in Fig. 1.

**Table 4:** Findings on MRI brain imaging in 35 patients with PRES

	Percentage (%)	Number of patients
Typical PRES pattern	80	28
Dominant parieto-occipital pattern	66	23
Holohemispheric watershed pattern	14	5
Isolated superior frontal sulcus pattern	0	0
Atypical PRES pattern	20	7
Cerebellar pattern	9	3
Frontal and temporal lobes pattern	6	2
Basal ganglia pattern	3	1
Splenium corporis callosi	3	1
Others		
Restricted diffusion	6	2
Enhancement	6	2
Haemorrhage	23	8

At discharge, eight patients had a good recovery (GOS 5), 16 a moderate disability (GOS 4), 11 a severe disability (GOS 3), and two patients had died (GOS 1). Twenty-five patients (68%) recovered completely from PRES to their premorbid status, and ten (27%) had residual symptoms when discharged from the hospital, mostly epileptic seizures or visual disturbances.

## Discussion

According to the algorithm of Fugate and Rabinstein [1], a patient with at least one acute neurological symptom, including seizures, encephalopathy/confusion, headache or visual disturbances and at least one risk factor, including severe hypertension/blood pressure fluctuations, renal failure, immunosuppressant therapy or chemotherapy, eclampsia or autoimmune disorder, should be suspected to have PRES on clinical grounds.

From our findings, we suggest that different presenting symptoms and risk factors should be given different weights. We found that seizures were the presenting symptom which best predicted PRES, followed by encephalopathy. Previous studies [6, 7, 10], have reported that epileptic seizures and encephalopathy were the two most frequent symptoms in patients with PRES. In addition, visual disturbances were also a good predictor of PRES, but headache and focal neurological deficits did not predict PRES in our study. Headache and focal neurological deficits have been reported to be less frequently associated with PRES than seizures or encephalopathy [6–8, 10]. We noted in our study that headache was present in a greater proportion of patients without PRES (44%) than patients with PRES (35%). We suggest, therefore, that headache could be omitted from the algorithm suggested by Fugate and Rabinstein [1].

Hypertension was the risk factor which best predicted PRES in our patients (31 patients, 84%), confirming the previous observations [6–9] reporting hypertension in 61–89% of patients with PRES. Our study further showed that chemotherapy and renal failure were valuable predictors of PRES. Data from the literature on chemotherapy and renal failure in patients with PRES are variable, with results ranging from 5 to 44% of patients with PRES receiving chemotherapy [6–10] and 12–57% of patients with PRES having renal failure [9, 10]. We also noted a significant association of an increasing number of risk factors with PRES. Autoimmune disorders were present in 16% of our patients with PRES, which is comparable with 8–45% of patients with PRES in previous reports. Also, immune suppressive therapy was given to 30% of patients with PRES in our study, which is similar to 23–43% of patients with PRES in previous reports [1, 7, 8, 10]. Autoimmune disorders and immune suppressive therapy, however, did not predict PRES in our patients. Our study design allows us to propose hypertension, chemotherapy and renal failure as risk factors predicting PRES, but not autoimmune disease or immune suppressive therapy. We identified epileptic seizures, encephalopathy, visual deficits, hypertension, chemotherapy and renal failure as the best clinical predictors for PRES. All patients with PRES had at least one of these predictors. When acute neurological symptoms and risk factors were considered separately, 5% of patients with PRES had no epileptic seizures, encephalopathy or visual deficits, and 8% had no hypertension, chemotherapy or renal failure. Eclampsia was present in only one patient of our study. For this reason, it was not possible to determine any relevant association with PRES. Brewer and colleagues [11] described the presence of PRES on MR imaging in 46 out of 47 women with eclampsia, suggesting that eclampsia is not a risk factor, but a manifestation of PRES.

A majority of patients (83%) in our study in whom PRES was considered in the differential diagnosis did not have MR confirmation of PRES. The most frequent differential diagnosis for PRES was primary or secondary headache (26%). Toxic-metabolic encephalopathy was the second most frequent differential diagnosis (21%). This can be explained by the high association of confusion with both PRES and toxic-metabolic encephalopathy. From our data, we agree with Fugate and Rabinstein [1] that the list of differential diagnoses of PRES is long and varied. Interestingly, the final clinical diagnosis was PRES in three patients despite normal imaging. We, therefore, agree that the diagnosis of PRES is a clinical diagnosis that may be confirmed by radiological findings, but may also exist without abnormalities on brain imaging. This is important, since it may influence clinical management. As long as there is no specific treatment for PRES, the only way to improve the outcome in patients with PRES is to establish the diagnosis as soon as possible, in order to treat the underlying causes. We emphasize the need for early correction of the risk factors which predicted PRES in our study, i.e. antihypertensive treatment, and when, possible, cessation of chemotherapy and treatment of renal failure.

The most frequent PRES pattern on neuroimaging in our patients was a typical parieto-occipital pattern, confirming previous observations. In our study, there was no patient with a dominant superior frontal sulcus pattern, contrary to results from Bartynski and Boardman [3] and Liman et al. [7], in which this pattern was found in 27 and 17%, respectively.

It is important for the clinician to be aware of less typical patterns as well, such as vasogenic oedema in the basal ganglia or brainstem, or lesions in the splenium corporis callosi, in order to establish a diagnosis of PRES. PRES is usually reversible and patients tend to make a good recovery. Two patients in our study, however, possibly died from PRES. Fifty % of patients had moderate or severe disability at the time of discharge from the hospital, but taking into account their premorbid status, 68% of patients had recovered completely from PRES at the time of discharge. These findings confirm that PRES is not always a reversible syndrome.

A weakness of our study was its retrospective nature. It is possible that acute neurological symptoms, such as visual disturbances or encephalopathy, may have been overlooked or not documented properly. On the other hand, we do not know how many patients fulfilling the clinical diagnostic criteria did not have MR brain imaging.

In conclusion, we identified epileptic seizures, encephalopathy, visual deficits, hypertension, chemotherapy and renal failure as best predictors of PRES. A clinical diagnosis of PRES should prompt MR imaging of the brain to confirm the diagnosis. In a minority of patients with a clinical diagnosis of PRES, MR of the brain may be normal. An early diagnosis of PRES may change medical and improve outcome management.

## Electronic supplementary material

Below is the link to the electronic supplementary material.

Supplementary material 1 (DOCX 14 kb)
