# Supplementary material for: Clinical predictors and differential diagnosis of posterior reversible encephalopathy syndrome
Source: Acta Neurol Belg. 2017 Jan 31;117(2):469–75. doi: 10.1007/s13760-017-0750-6 (PMC5440491; doi:10.1007/s13760-017-0750-6)
Supplement: Supplementary file 1 — Supplementary material 1 (DOCX 14 kb) [file 13760_2017_750_MOESM1_ESM.docx]

| Differential diagnosis | Number of patients (total = 183) |
| --- | --- |
| Headache | 47 patients |
| primary headache (migraine or tension-type headache) | 35 patients |
| secondary headache (hypertensive headache, medication-induced headache, headache caused by pre-eclampsia, post-puncture headache) | 12 patients |
| Metabolic-toxic encephalopathy | 38 patients |
| metabolic encephalopathy, | 28 patients |
| toxic encephalopathy, | 7 patients |
| post-anoxic encephalopathy, | 2 patients |
| benzodiazepines withdrawal | 1 patient |
| Vascular pathology | 22 patients |
| ischemic stroke | 18 patients |
| venous sinus thrombosis | 1 patient |
| parenchymal haemorrhage | 1 patient |
| ischemic stroke or PRES | 1 patient |
| chronic subdural hematoma | 1 patient |
| Infectious pathology | 15 patients |
| infectious encephalitis or meningitis | 10 patients |
| systemic infection | 5 patients |
| Epileptic seizures not caused by PRES | 14 patients |
| acute symptomatic epileptic seizure | 11 patients |
| status epilepticus | 2 patients |
| epileptic seizure secondary to an old cerebral infarction | 1 patient |
| Neuro-degenerative processes | 8 patients |
| Psychiatric disease | 8 patients |
| multifactorial delirium | 3 patients |
| anxiety disorders | 2 patients |
| hyperventilation | 1 patient |
| a psycho-organic illness | 1 patient |
| psychiatric decompensation | 1 patient |
| Tumoral processes | 7 patients |
| malignant tumours | 6 patients |
| meningioma | 1 patient |
| Ophthalmological illness | 6 patients |
| cataract | 1 patient |
| idiopathic papilledema | 1 patient |
| choroid infarction | 1 patient |
| hypertensive retinopathy | 1 patient |
| amaurosis fugax | 1 patient |
| aspecific visual loss | 1 patient |
| Clinical diagnosis of PRES | 3 patients |
| Inflammatory disorders | 3 patients |
| autoimmune encephalitis, | 1 patient |
| neuro-Behçet’s disease | 1 patient |
| neuro-sarcoïdosis | 1 patient |
| Disorders affecting intracranial pressure | 3 patients |
| secondary pseudotumor cerebri due to obstructive sleep apnea syndrome | 1 patient |
| liquor hypotension | 1 patient |
| hydrocephalus | 1 patient |
| Others | 5 patients |
| vasovagal syncope | 1 patient |
| subacute cryptogenic meningo-encephalitis | 1 patient |
| peripheral vestibular disease | 2 patients |
| isolated nystagmus | 1 patient |
| Exact diagnosis unknown (4 patients, 2%) |  |

Table 5 (supplemental). Differential diagnoses in 183 patients without radiological diagnosis of PRES.
